# Synthesis of new type of Au-magnetic nanocomposite and application for protein separation thereof

**DOI:** 10.1186/1556-276X-7-369

**Published:** 2012-07-03

**Authors:** Yu Song, Ling Tao, Xiangchun Shen

**Affiliations:** 1 Department of Pharmacy, Third Affiliated Hospital of Southern Medical University, Guangzhou 510630, China; 2 Research Division of Pharmacology, Guiyang Medical College, Guiyang 550004, China

**Keywords:** Gold, Magnetic, Bifunctional, Protein separation, Nanoparticle

## Abstract

We present a different strategy for synthesizing the Au-γ-Fe_2_O_3_ bifunctional nanoparticle by using a larger (50 nm) Au nanoparticle as the core surrounded by smaller (10 nm) γ-Fe_2_O_3_ nanoparticles. The synthesis of the composite nanoparticles is quite facile based on a simple redox process whereby Fe^2+^ is used to reduce Au^3+^. The morphology and composition of the product is measured by transmission electron microscopy, X-ray powder diffraction and UV–vis spectroscopy. We demonstrate the utility of these as-prepared Au-γ-Fe_2_O_3_ nanoparticles by showing they can be used to separate proteins in solution. For example, bovine serum is efficiently removed from an aqueous solution with the simple addition of the NPs and application of a small magnet. Sodium dodecyl sulfate-polyacrylamide gel electrophoresis is performed to evaluate the fidelity and efficiency of the protein separation procedure.

## Background

Nanoparticles (NPs) containing two completely different elemental compositions (i.e., bifunctional nanomaterials) enable a single particle to have physical properties vastly superior to those made solely from the individual elements. Due to their increased versatility, such bifunctional nanomaterials have enhanced potential for the development of new applications in many different areas, especially in biotechnology. For example, a single composite nanoparticle derived from gold (Au) and iron oxide nanoparticle subunits is quite versatile, having excellent surface chemistry, superior optical characteristics of gold and superparamagnetic properties of iron oxide [1-7].

Commonly, such Au-maghemite (γ-Fe_2_O_3_) bifunctional nanoparticles have a γ-Fe_2_O_3_ core, either a solid Au shell or smaller Au nanoparticles surrounding the core [8,9]. We present a different strategy for synthesizing the Au-γ-Fe_2_O_3_ bifunctional nanoparticle by using a larger (50 nm) Au nanoparticle as the core surrounded by smaller (10 nm) γ-Fe_2_O_3_ nanoparticles. The synthesis of the composite nanoparticles is quite facile based on an easy redox process whereby Fe^2+^ was used to reduce Au^3+^. One advantage of this composition is that the size of the bifunctional nanoparticle is easily tuned by changing the size of the Au nanoparticle core while still maintaining a strong magnetic response since a significant amount of magnetic material composes the single particle.

We demonstrate the utility of these as-prepared Au-γ-Fe_2_O_3_ nanoparticles by showing they can be used to separate proteins in solution. For example, bovine serum is efficiently removed from an aqueous solution with the simple addition of the NPs and application of an external magnetic field. Sodium dodecyl sulfate-polyacrylamide gel electrophoresis (SDS-PAGE) is performed to evaluate the fidelity and efficiency of the protein separation procedure.

## Methods

All chemicals were purchased from Sigma-Aldrich Corporation (MO, USA) and used as received without further purification. Deionized water was used throughout. The TEM images were taken using a JEOL 2000EX transmission electron microscope (JEOL Ltd., Tokyo, Japan) at an accelerating voltage of 200 kv. The UV–vis spectra were taken by Lambda 950 UV–vis spectrometer (PerkinElmer, MA, USA).

**Figure 1:**
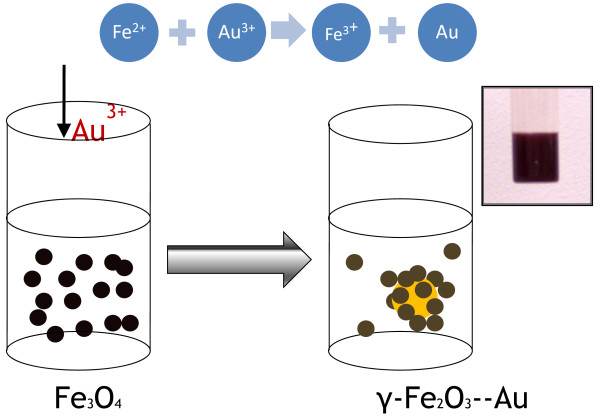
Schematic of the formation of maghemite-gold bifunctional nanomaterials.

In this method, magnetite nanoparticles were prepared first in water solvent by the chemical precipitation method. Then, gold precursor was added in the solution and reduced by Fe^2+^ which was oxidized to Fe^3+^. Magnetite change to maghemite and attach on gold nanoparticles (as shown in Figure 1). In a typical process, a mixture of 0.1 mmol of FeCl_2_·3H_2_O, 0.2 mmol of FeCl_3_·6H_2_O and 0.1 mmol D-lysine in 50 ml deionized water was stirred and bubbled with N_2_ for 30 min, and then, 0.6 ml of 5 N ammonium was added in the mixture under N_2_ protection. The color of the mixture changed to black immediately while the mixture was continually stirred for another 30 min and added with 2 ml of 0.05 M HAuCl_4_ aqueous solution drop by drop into the black mixture. The color changed to purple black slowly while on continuous stirring for 60 min. Separated by external magnate, the liquid was almost colorless, and the paste was purple black which was washed by deionized water and separated by external magnate three times. The final product, which was purple black solution, was redispersed in deionized water for further measurement. Figure 1 shows the procedure of formation of maghemite-gold bifunctional nanomaterials. Inset photo is the final product in water, which shows the purple-black solution.

**Figure 2:**
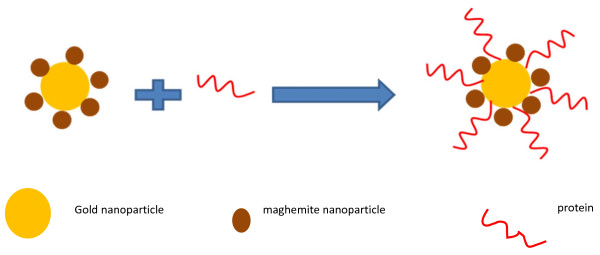
Schematic of the protein separation by the nanocomposite.

Protein separation is one of the basic applications of this kind of bifunctional nanomaterials. To use these materials for protein separation, the sample was washed several times to make sure there is no free gold nanoparticle in the solution, and bovine serum solution was added in the sample. Then, by using external magnetic separation, the sample was divided to liquid and paste, which was redispersed in water, for SDS-PAGE electrophoresis gel stained by Coomassie blue. Figure 2 shows the mechanism for protein separation by the bifunctional nanocomposite.

## Results and discussion

**Figure 3:**
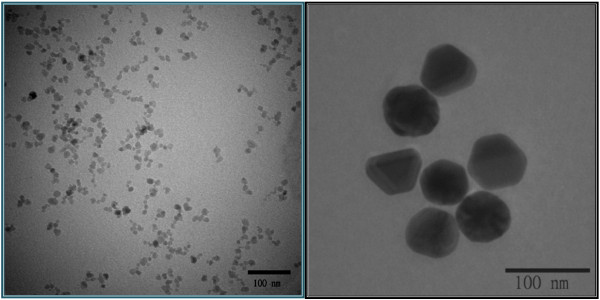
TEM images for 10-nm magnetite (left) and 50-nm gold (right) nanoparticles.

**Figure 4:**
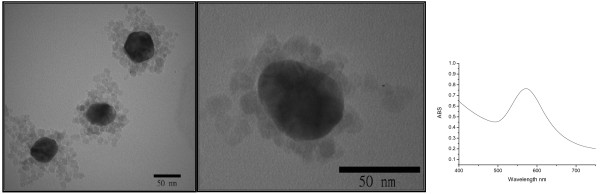
TEM images for 10-nm maghemite attached on 50-nm gold nanoparticles and UV–vis spectrum.

To compare with the bifunctional nanocomposite, gold (50 nm) and magnetite (10 nm) nanoparticles were synthesized and characterized separately. From TEM images and AFM image (Figure 3), the morphology of Fe_3_O_4_ (magnetite) and Au nanoparticle is shown clearly. After the addition of gold precursor to the magnetite solution, the structure of 50-nm gold nanoparticle surrounded by 10-nm maghemite nanoparticles was formed (Figure 4). Due to this kind of structure, the UV peak for gold shifted to 565 nm.

**Figure 5:**
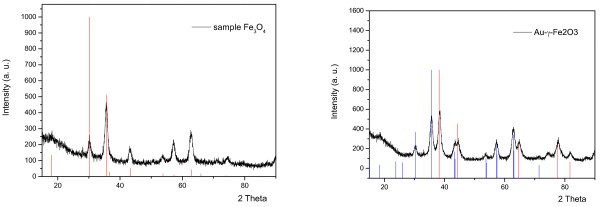
**XRD patterns of Fe**_**3**_**O**_**4 **_**(left) and Au-γ-Fe**_**2**_**O**_**3 **_**(right).**

The composition of the synthesized materials was identified by XRD, as shown in Figure 5. The left pattern is the data for magnetite (Fe_3_O_4_) (black line in left figure), which is before the addition of Au precursor, and the right pattern is the data for Au-γ-Fe_2_O_3_ composite (black line in right figure). The results match the data in the 2003 JCPDS-International Centre for Diffraction Data for magnetite (89–0951) (red line in left figure), maghemite (89–5894) (blue line in right figure) and gold (89–3697) (red line in right figure). These results can provide two facts: first is after magnetic separation; the gold is still in the sample. Second is that magnetite was reduced to maghemite.

To test these materials for protein separation, bovine serum solution was added in the sample. Then, by using external magnetic separation, the sample was divided to liquid and paste which was redispersed in water for SDS-PAGE electrophoresis gel.

**Figure 6:**
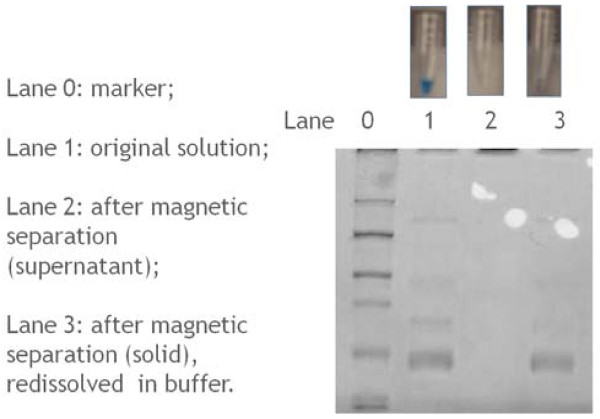
**Bradford protein assay of the separation efficiency (top photos).** SDS-PAGE analysis of the separated protein: lane 0, marker; lane 1, original protein and particle mixture solution; lane 2, supernatant part after separation; lane 3, solid part after separation, which was redissolved in buffer.

The Bradford protein assay protocol (Coomassie Blue G-250) was used as an instant method to examine for the existence of protein in those two parts of sample [10]. If a certain part contains proteins, it will turn the originally brown Coomassie Blue G-250 solution into a blue color, while the part without protein in it will leaves Coomassie Blue G-250 brown. As shown in the above photos of Figure 6, sample 1 is the original sample solution which was before the magnetic separation. It contained protein since the color of Coomassie Blue solution was turned into blue. Sample 2 is the supernatant part after magnetic separation. It contained no protein since the color of Coomassie Blue solution remained brown. Sample 3 is the solid part after magnetic separation which was redissolved in buffer. It contains proteins since it turned the solution into blue.

Sodium dodecyl sulfate- polyacrylamide gel electrophoresis was then applied according to the literature [11]. As shown in Figure 6, compared to the marker in lane 0, the original mixture solution (as shown in lane 1) contained proteins of various molecular sizes. After magnetic separation, those proteins were removed from the solution (lane 2). For the solid sample after separation, the proteins were obtained again (lane 3), so the protein was effectively separated and collected.

## Conclusions

We reported the synthesis of gold-maghemite nanoparticles and their use in separating proteins. The as-prepared nanocomposites combined the merits of both gold and magnetic nanoparticles, and were produced by a very easy method. Furthermore, this experiment has also suggested a new way to synthesize various bifunctional or multifunctional composite nanomaterials through simple redox process.

## Competing interests

The authors declare that they have no competing interests.

## Authors' contributions

YS carried out the synthesis, TEM and XRD measurements. LT carried out Bradford protein assay and SDS − PAGE, and participated in the design of the study. XS conceived of the study, participated in its design and coordination, and drafted the manuscript. All authors read and approved the final manuscript.

## Authors' information

YS is a an MD and a pharmacist-in-charge. YS's research areas are pharmaceutics and Chinese medicine pharmacology, and is affiliated to Department of Pharmacy, Third Affiliated Hospital of Southern Medical University, Guangzhou, 510630, China. LT is an MD whose research is on pharmaceutics and Chinese medicine pharmacology, and is affiliated to the Research Division of Pharmacology, Guiyang Medical College, Guiyang, 550004, China. XS is also an MD and PI whose research areas are on nanotechnology, pharmaceutics and Chinese medicine pharmacology. XS is affiliated to the Research Division of Pharmacology, Guiyang Medical College, Guiyang, 550004, China.
